# Incidence of Urinary Infections and Behavioral Risk Factors

**DOI:** 10.3390/nu16030446

**Published:** 2024-02-02

**Authors:** Magdalena Mititelu, Gabriel Olteanu, Sorinel Marius Neacșu, Iuliana Stoicescu, Denisa-Elena Dumitrescu, Emma Gheorghe, Monica Tarcea, Ștefan Sebastian Busnatu, Corina-Bianca Ioniță-Mîndrican, Ovidiu Tafuni, Ionela Belu, Antoanela Popescu, Sergiu Lupu, Carmen Elena Lupu

**Affiliations:** 1 Department of Clinical Laboratory and Food Safety, Faculty of Pharmacy, Carol Davila University of Medicine and Pharmacy, 020956 Bucharest, Romania; magdalena.mititelu@umfcd.ro (M.M.); gabriel.olteanu@mst.umfcd.ro (G.O.); 2 Department of Pharmaceutical Technology and Bio-Pharmacy, Faculty of Pharmacy, Carol Davila University of Medicine and Pharmacy, 020945 Bucharest, Romania; 3 Department of Chemistry and Quality Control of Drugs, Faculty of Pharmacy, Ovidius University of Constanta, 900470 Constanta, Romania; iuliana.stoicescu@univ-ovidius.ro; 4 Department of Organic Chemistry, Faculty of Pharmacy, Ovidius University of Constanta, 900470 Constanta, Romania; denisa.dumitrescu@univ-ovidius.ro; 5 Department of Preclinical Sciences I—Histology, Faculty of Medicine, Ovidius University of Constanta, 900470 Constanta, Romania; 6 Department of Community Nutrition and Food Safety, G.E. Palade University of Medicine, Pharmacy, Science, and Technology of Targu Mures, 540142 Mures, Romania; monica.tarcea@umfst.ro; 7 Department of Cardio-Thoracic Pathology, Faculty of Medicine, Carol Davila University of Medicine and Pharmacy, 050474 Bucharest, Romania; stefan.busnatu@umfcd.ro; 8 Department of Toxicology, Faculty of Pharmacy, Carol Davila University of Medicine and Pharmacy, 020945 Bucharest, Romania; corina-bianca.ionita-mindrican@drd.umfcd.ro; 9 Department of Preventive Medicine, Nicolae Testemițanu State University of Medicine and Pharmacy from the Republic of Moldova, MD-2004 Chisinau, Moldova; ovidiu.tafuni@usmf.md; 10 Department of Pharmaceutical Technology, Faculty of Pharmacy, University of Medicine and Pharmacy of Craiova, 200638 Craiova, Romania; ionela.belu@umfcv.ro; 11 Department of Pharmacognosy, Faculty of Pharmacy, Ovidius University of Constanta, 900470 Constanta, Romania; antoanela.popescu@univ-ovidius.ro; 12 Department of Navigation and Naval Transport, Faculty of Navigation and Naval Management, Mircea cel Batran Naval Academy, 900218 Constanta, Romania; sergiu.lupu@anmb.ro; 13 Department of Mathematics and Informatics, Faculty of Pharmacy, Ovidius University of Constanta, 900001 Constanta, Romania; clupu@univ-ovidius.ro

**Keywords:** recurrence of urinary infections, healthy diet, functional foods, public health

## Abstract

This evaluation of the impact of behavioral risk factors on the incidence of urinary infections was based on a questionnaire in which 1103 respondents, predominantly women (883), participated. From the statistical processing of the data, it was observed that 598 of the respondents were of normal weight; the rest, more than half, were underweight or overweight (χ^2^ = 32.46, *p* < 0.001), with male respondents being predominantly overweight or obese (169 out of a total of 220). Most of the respondents were young (χ^2^ = 15.45, *p* < 0.001), under the age of 45 (840). According to the processed data, it was found that respondents in the age group of 26–35 years showed the greatest vulnerability to recurrent urinary infections, while the age group of 18–25 years recorded the highest number of responses related to the rare presence or even absence of episodes of urinary infections. A body weight-related vulnerability was also noted among the respondents; the majority of obese people declared that they face frequent episodes of urinary infections. Regarding diet quality, 210 respondents reported an adherence to an unhealthy diet, 620 to a moderately healthy diet, and 273 to a healthy diet. Of the respondents who adhered to a healthy diet, 223 were women (χ^2^ = 2.55, *p* = 0.279). There was a close connection between diet quality and the frequency of urinary infections: from the statistical processing of the data, it was observed that the highest percentage of respondents who rarely (57.14%) or never got urinary infections (29.30%) were among those who adhered to a healthy diet, and the highest percentage of those who declared that they often got urinary infections were among those with increased adherence to an unhealthy diet (χ^2^ = 13.46, *p* = 0.036). The results of this study highlight a strong impact of obesity, reduced consumption of fruit and vegetables, and sedentary lifestyle on the risk of recurring urinary infections.

## 1. Introduction

The pathology of urinary infections shows an upward trend and can significantly affect quality of life and even pose the risk of premature death due to the complications it produces in the human body, especially when the management of risk factors is insufficient, preventive measures are ignored, or when treatment regimens are not applied correctly.

Globally, it is estimated that more than 404.6 million individuals [1] were diagnosed with urinary tract infections (UTIs) in 2019. This situation is constantly increasing: if we refer to statistics from 1990, 252.25 million cases were detected then [1]. So, we are facing an increase of more than 50% (60.40%, to be exact) in terms of cases of UTIs [2].

Urinary tract infections are bacterial infections that affect any part of the urinary tract (bladder and/or kidneys) [3]. The most common infection is with Escherichia coli, but there are other pathogenic microorganisms involved, such as *Klebsiella pneumoniae*, *Pseudomonas aeruginosa*, and *Proteus*. Among Gram-positive uropathogens, *Enterococcus* spp. and *Staphylococcus* spp. are involved [4,5]. In a study that analyzed 1588 urine samples from female patients, *Escherichia coli* was detected as the most common uropathogen (58.37%) [5].

Knowing the risk factors that predispose to infections with uropathogenic microorganisms is the key to success in the primary prevention of UTIs. Risk factors include an unfavorable family history, the female sex (anatomically, women have a shorter urethra), advanced age (increased incidence in older women is caused by altered health status (diabetes, neurogenic bladder, recent catheterizations, urinary tract obstructions, chronic kidney disease)), frequent sexual activity or with different partners, a suppressed immune system (in case of pre-existing HIV infection), use of the diaphragm or spermicides as a contraceptive method, and anatomical or hormonally induced changes in menopausal patients. In the case of men, prostate adenoma (benign prostatic hyperplasia) is the main anatomical structural change that predisposes to UTIs [3,6,7]. It is also important to sound the alarm on the injudicious use of antibiotics, which disrupts the balance of the natural microflora and causes an increase in the resistance of pathogens to antimicrobial agents, with significant risks in terms of the optimal management of infections [8].

However, there are many situations where primary prevention fails and the patient is eventually diagnosed with a UTI. The therapeutic approach is essential, because even an optimal antibiotic treatment can take several days to treat the symptoms. Left untreated or without proper treatment, the complications of UTIs are life-threatening. These include pyelonephritis (emphysematous pyelonephritis and emphysematous cystitis), which may progress to sepsis; permanent kidney damage, with progression to renal dysfunction, hypertension and renal failure; and in the case of pregnant mothers, it can increase the risk of premature birth and low birth weight of the fetus [6,7,9,10]. Other complications of UTIs are persistent lower urinary tract symptoms, chronic prostatitis, prostatic abscess, and urinary incontinence [10].

In the case of UTIs, there is a high probability of recurrence. This can be caused either by bacterial persistence (infection with the same microorganism coming from another region of the urinary tract) or by reinfection (infection with a different bacterium or the same uropathogen coming from outside the urinary tract) [11]. Recurrence refers to the occurrence of more than two symptomatic episodes within 6 months or more than three symptomatic episodes within a 12-month interval [7]. Recurrences are caused by multiple risk factors, such as changes in the vaginal bacterial microflora, frequent sexual activity (more than four times a week), multiple sexual partners, use of spermicides that affect vaginal pH, lack of urination after intercourse, vaginal douching, uncomfortable underwear, poor or faulty hygiene, as well as family history or personal history of childhood UTIs [12,13,14,15]. For women who are postmenopausal, a history of UTIs during premenopause increases the risk of recurrence. Of course, with advancing age, there appear various body imbalances that considerably increase the risk of recurrence, namely vulvovaginal atrophy, urinary incontinence, anterior vaginal wall prolapse, intermittent or permanent urinary catheterization procedures, as well as pathologies such as chronic kidney disease or type 2 diabetes [7,16,17,18,19].

Similar to the risk factors for the occurrence of UTIs, it is important to know the factors that predispose to recurrences in order to implement preventive measures and lifestyle, hygiene, and dietary changes that have the role of reducing the risk of recurrences and especially of complications.

Diet and lifestyle can influence the predisposition to urinary infections. In terms of lifestyle, personal hygiene, rest, and physical activity especially influence the body’s immunity and detoxification capacity. Proper nutrition and hydration of the body also play an essential role in the prevention and improvement of urinary infection pathologies [20,21,22,23].

**Figure 1:**
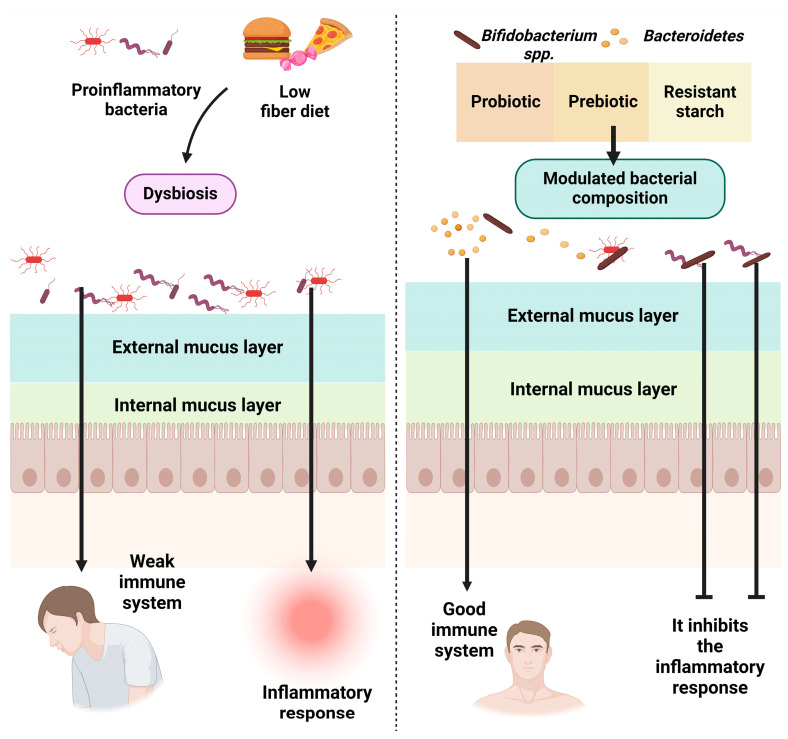
The influence of food on the intestinal microbiome. Created with BioRender.com (accessed on 25 October 2023).

Foods, especially functional ones, bring a supply of valuable nutrients and protective factors for the body (vitamins, mineral salts, antioxidants, fibers, probiotics). Most biocompounds with therapeutic potential are found in fruits, vegetables, and other therapeutic plants. Medicinal plants are known, but there is also a series of vegetables and fruits which are rich in phytonutrients that can prevent or improve urinary infections [24,25,26]. Prebiotics and probiotics are also important for balancing the microbial flora in the intestinal microbiome [27,28] and have an immune-stimulating and anti-inflammatory effect (Figure 1). On the other hand, ultra-processed foods, which are poor in nutrients and rich in food additives, and foods contaminated with various toxic substances (heavy metals, pesticides, microplastics) affect immune function, the activity of the intestinal microbiome, liver function, and the body’s detoxification capacity, and favor inflammatory processes [29,30,31,32].

**Figure 2:**
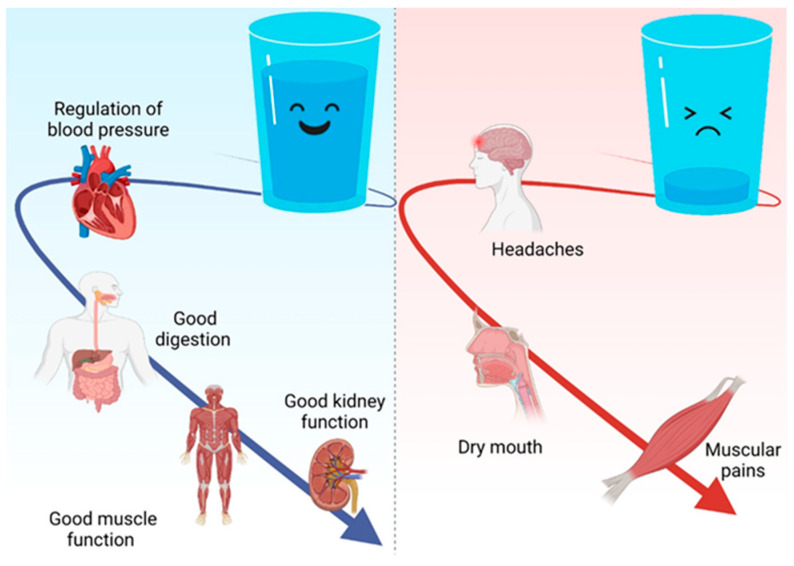
The role of water in the body. Created with BioRender.com (accessed on 25 October 2023).

Water is vital for the proper functioning of nerve cells, for the regulation of blood pressure, for optimal digestion and absorption of nutrients from the digestive tract, and for the functioning of the kidneys, helping to eliminate waste and excess nutrients through urine (Figure 2). Also, water has a lubricating action on the joints; it helps with the formation of synovial fluid and the proper functioning of the muscles. In addition, water provides the body with some of the minerals lost through sweat [33,34,35,36].

As previously presented, there are numerous risk factors that favor the recurrence of urinary infections, and their poor management can lead to serious complications and even premature death. Many factors are dependent on diet and lifestyle, and a balanced diet, the choice of food sources in such a way that they are as uncontaminated or industrially processed as possible [37,38], and the adoption of a healthy lifestyle that includes correct hygiene of the body [39], effective hydration, avoiding excess alcohol, avoiding tobacco [40] or drug use, effective rest, and avoiding sedentarism represent important aspects that can beneficially influence our immunity and resistance to infections. In light of these important aspects, an assessment of the impact of behavioral risk factors on the frequency of urinary infections was conducted. In the case of the influence of diet quality, the respondents’ adherence to a healthy diet was also evaluated, depending on the frequency of consumption of nutritious foods rich in protective factors for the body (fruit and vegetables, whole grains, non-hydrogenated vegetable fats, fish, seafood, dairy products, eggs, meat) but also the frequency of consumption of foods from the category of junk food products that are recommended to be avoided (packaged sweets, hot dogs, chips, hamburgers, fries, pastries, sweetened drinks, energy drinks). In parallel, the distribution of infectious episodes was analyzed with respect to a series of individual characteristics of the respondents (sex, age, body mass index). The identification of the most aggressive behavioral risk factors underlying recurrent urinary infections allows for a more efficient management of prevention methods.

The analysis of the impact of behavioral risk factors on the incidence of urinary infections was based on a questionnaire designed to analyze the frequency of urinary infections associated with the dietary habits and lifestyle of the respondents. The evaluation of some aspects related to the treatment of urinary tract infections was followed by questions that included the medicinal substances used, supplements used in the treatment, the frequency of medical control, the use of a treatment prescribed by a doctor or not, and the use of antimicrobial tests when administering antibiotics, because the approach to drug therapy can also influence the recurrence of infectious episodes.

## 2. Materials and Methods

### 2.1. Study Design

In order to evaluate the frequency of urinary infections correlated with behavioral risk factors, a cross-sectional observational study was carried out, based on a questionnaire with 39 items that included the collection of demographic and anthropometric data of the respondents (age, sex, residence, education level, occupational status, height, weight, frequency of urinary infections, causes of urinary infections, associated symptoms, eating habits and the amounts consumed from different food groups, water consumption, consumption of alcoholic beverages, type of diet, presence of the habit of smoking and its frequency, presence of physical activity and its frequency, natural and medicinal treatments used for urinary infections, carrying out periodic specialized check-ups, carrying out antibiograms in the case of antibiotic treatment, existing associated pathologies). For a more precise analysis of eating habits, the following factors were taken into account: the daily consumption of portions of fruit and vegetables (multiples of 100 g); portions of alcoholic beverages (125 mL = glass of wine or 50 mL = portion of spirits) or sweetened beverages (330 mL = one glass); daily water consumption; frequency of consumption (daily, more than twice a week, twice a week, once a week, very rarely or not at all) of food products from different groups (fruit and vegetables, dairy, pasta, cereals, meat, eggs, fish, seafood, sweets, pastries, alcoholic and sweetened beverages, fast food products).

The questionnaire was designed to collect data on demographic information, anthropometric measurements, urinary infection history, associated symptoms, dietary habits, and various lifestyle factors. The questions were formulated based on the existing literature, expert opinions, and consideration of the study population.

Between July and August 2023, the questionnaire was distributed using Google Forms via social media, WhatsApp (https://www.whatsapp.com, accessed on 1 July 2023), and institutional emails within participating higher education institutions or companies. The questionnaire was distributed alongside a short text in which the respondents were assured about the protection of personal data and anonymous participation in the study but were also asked to answer the questions honestly in order not to affect the quality and correctness of the study. Mainly, employees and students of three university centers from different areas of Romania (Bucharest, Craiova, and Constanta) participated in this study. Teaching staff instructed students to complete the questionnaire as correctly as possible and asked them to disseminate it to their family members and their groups of friends. The aim was to gather voluntary responses from individuals aged 18 and above, with no discrimination based on gender, religion, or political views. To maintain confidentiality, the questionnaire was designed to collect data anonymously, without gathering personal identifiers like email addresses. Participants provided informed consent before completing the questionnaire, adhering to confidentiality standards. Exclusion criteria: age under 18, resident outside Romania. The group of respondents was varied, with different degrees of knowledge in the medical field. This study was approved by the Ethics Commission of the University of Medicine and Pharmacy in Craiova, approval number 158, dated 4 June 2023.

A team of 6 experts with diverse backgrounds in urology, nutrition, and questionnaire development assisted in validating the questionnaire, subsequently testing it during a pilot phase involving 170 participants. This phase aimed to refine the questionnaire, enhancing its accuracy and clarity. To evaluate its internal consistency, the Cronbach’s α coefficient was computed, resulting in a final value of 0.86, indicating a high level of internal consistency for the validated questionnaire [41,42]. In order to ensure an efficient testing of the questionnaire in the pilot phase, a number of answers of 10–15% of the sample of respondents included in the study is necessary. Testing in the pilot phase was necessary to identify potential issues such as ambiguities, redundancies, or difficulties in comprehension. Adjustments were made accordingly to enhance clarity and relevance.

### 2.2. Sample Size Determination

The sample size for this study was determined by the Cochran formula [43] for populations with unknown disease prevalence which allows for obtaining a sample representative of larger populations. The minimum sample size required according to Cochran’s formula was 398 (with an error of ±5% and a confidence level of 95%).

The aim was to obtain a study with a maximum error of ±4%. According to the most recent census in Romania, the number of people over 18 reported on 1 December 2021 is 15,330,990 [44]. Thus, the minimum required sample size for our study was 601 (for a 95% confidence interval and ±4% error).

### 2.3. Sample Size Pilot Study

Whitehead et al. [45] stated that for a pilot study, at least 30 respondents are required. Furthermore, Julious [46] concluded that 12 participants are sufficient to obtain a credible mean and variance. Connelly (2008) [47] suggested that the sample for a pilot study should be a minimum of 10% of the designed sample for the main study.

In this paper, the formula provided by Viechtbauer et al. [48] to calculate the sample size required for a pilot study was applied, using a 95% confidence interval and an error of ±4%. This resulted in a required sample size of 74 to allow for the detection of feasibility issues. Considering the possible uncertainty and the variability of eating styles in the different geographical areas of Romania, 170 respondents were recruited.

For the pilot study, exploratory factor analysis was performed. The Kaiser–Meyer–Olkin (KMO) [49] statistic was 0.827, which confirms the suitability of the data for exploratory factor analysis [50], and the result of Bartlett’s test of sphericity [51] was *p* < 0.001, which indicates the absence of multicollinearity [51].

### 2.4. Questionnaire Validation

Following the dissemination of the questionnaire, 1033 valid responses were obtained (confidence interval of 95% and an error of ±3.05%).

To measure the internal consistency and reliability of the questionnaire, the Cronbach α coefficient, which is based on a linear model of correlation, was calculated. The value of the Cronbach α coefficient varies between 0 and 1. For an instrument to be considered consistent, it must reach a value as close to 1 as possible, with 0.70 being accepted as the cut-off point by most researchers [46].

For our questionnaire, the value of the Cronbach α coefficient is 0.86, indicating a high level of internal consistency.

### 2.5. Statistical Analysis

Data processing began with descriptive statistics to provide an overview of the participants’ baseline characteristics. The categories of gender, region of residence, and education level were presented using absolute frequencies and percentages, while continuous variables such as age, weight, and height were expressed as means and standard deviations.

Categorical variables were organized in contingency tables. The chi-square test was used to evaluate the impact of sex, age, BMI, adherence to a healthy diet, and physical activity on the frequency of urinary infections. The chi-square test was also used to determine the association between the frequency of urinary infections and the therapeutic approach.

An additional approach was to apply correspondence analysis (CA) to investigate the relationship between the frequency of UTIs and their causes. CA provided a visual representation of the associations between the nominal variables, providing a clear and intuitive insight.

To evaluate the influence of dietary habits on the frequency of urinary infections, multinomial logistic regression was performed. This involved introducing the outcome variable “adherence to a healthy diet” and examining the influence of independent variables such as gender, age, area of residence, education level, BMI, and frequency of urinary tract infections. The results were expressed as odds ratio (OR), confidence interval (95% CI), and associated *p* values.

Descriptive statistics, correspondence analysis, and multinomial regression analysis were performed using XLSTAT (version 2020, Addinsoft, New York, NY, USA). Statistical Package for Social Science, version 23 (SPSS Inc., Chicago, IL, USA), was used for exploratory factor analysis, to validate the questionnaire, and ensure its reliability. *p*-values of less than 0.05 were considered statistically significant.

## 3. Results

### 3.1. Socio-Demographic Characteristics

The questionnaire disseminated in this study registered a total number of 1103 valid answers, which came from 80.1% (883) female respondents and 19.9% (220) male respondents. BMI was calculated with the Quetelet equation [body mass (kg)/height (m^2^)] and interpreted according to the criteria of the World Health Organization [52,53]. After processing anthropometric data, it was observed that 46.6% (598) of the respondents were of normal weight, the majority of these being women (547), while male respondents were predominantly overweight (48.6% (107)) and 28.18% (62) of them were obese (χ^2^ = 32.46, *p* < 0.001). Regarding the age of the respondents, 76.2% were young, under 45 years old (χ^2^ = 15.45, *p* < 0.001). Table 1 displays the socio-demographic and anthropometric traits of the participants. As expected, most respondents were female, since urinary infections are more common among women. Regarding the area of residence, the majority of respondents came from urban environments (79.15%). This is probably also due to the fact that the urban population is more active online (χ^2^ = 10.089, *p* = 0.001). Over 80% of the study participants had postsecondary or higher education (χ^2^ = 15.529, *p* = 0.004). Over 69% of respondents commuted to work daily or worked in a hybrid mode, 2.81% worked remotely, and the rest were students, pensioners, or were unemployed (χ^2^ = 17.87, *p* = 0.013).

### 3.2. Frequency and Causes of Urinary Infections

**Figure 3:**
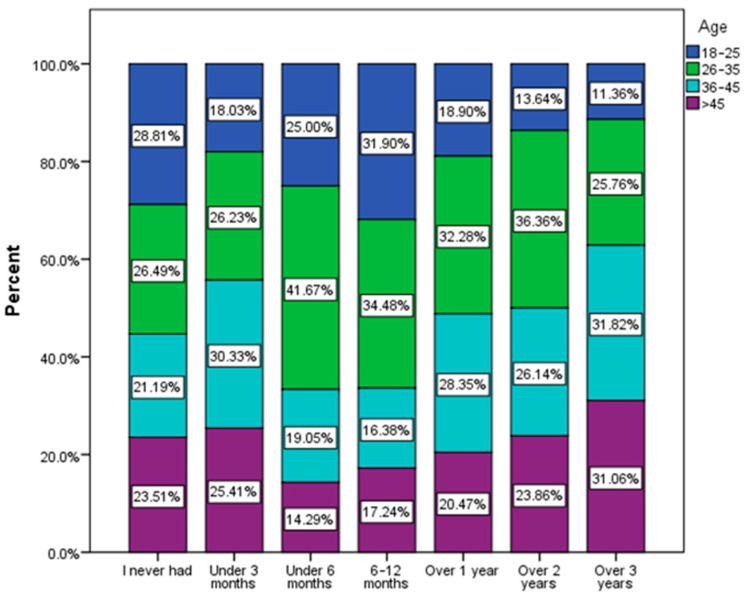
Last urinary infection episode by age group (χ^2^ = 64.91, *p* < 0.001).

The age group most vulnerable to urinary infections among the group of respondents was 26–35 years old. This group included the highest percentage of respondents who had urinary infections in the last 6 months (41.6%), in the last 6–12 months (34.48%), in the last year (32.28%), and in the last 2 years (36.36%). On the contrary, the lowest trend can be observed in the 18–25 age group according to the responses recorded (Figure 3). This age group (18–25) recorded the highest percentage of people who declared that they never had urinary infections (28.81%).

**Figure 4:**
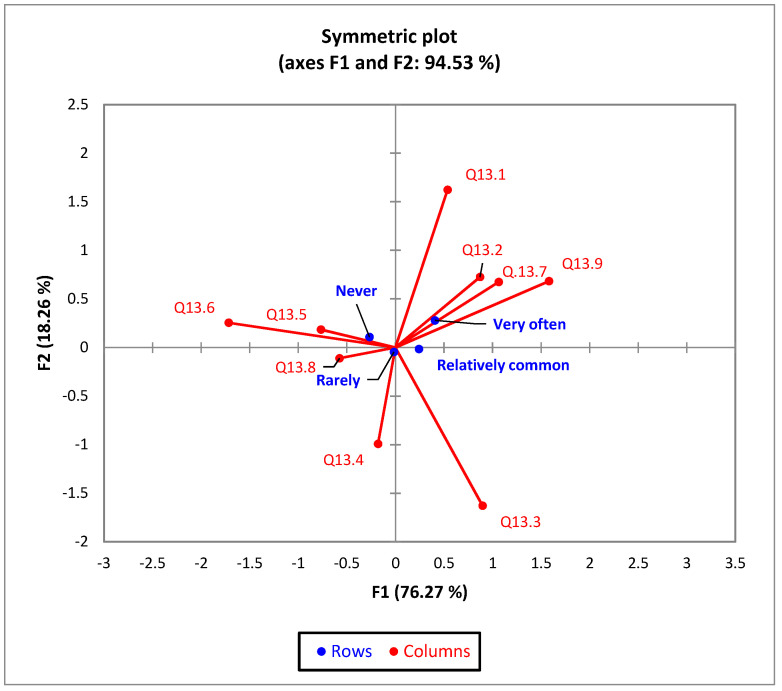
Frequency and main causes of urinary infections. F1 (frequency of urinary infections) and F2, represented by the following: Q13.1, sexual contact; Q13.2, constipation; Q13.3, exposure to cold; Q13.4, exposure to contaminated public spaces; Q13.5, exposure to contaminated spaces in healthcare settings (hospitals, clinics, doctors’ offices); Q13.6, improper hygiene; Q13.7, kidney stones; Q13.8, surgery; Q13.9, other causes.

In terms of the frequency of urinary infections and their main causes, the bi-plot indicates that 94.53% of the variability observed can be attributed to these two main components (F1 (76.27%) and F2 (18.26%)) (Figure 4). F1 represents the frequency of urinary infections and F2 the main causes of urinary infections.

Respondents who had severe urinary infections often believed that the main causes were sexual contact, constipation, and kidney stones.

### 3.3. Assessment of Behavioral Risk Factors

The next variable, “adherence to a healthy diet”, was introduced by coding the answers to questions aimed at quantifying the frequency of consumption of food products from different groups. The frequency of consumption of some nutritious food groups (vegetables, fruits, whole grains, virgin and extra virgin vegetable oils, eggs, fish and seafood, sources of probiotics such as fermented dairy products and mature cheeses, and amount of water consumed daily) was classified as a healthy diet. The unhealthy diet category included the frequency of consumption of saturated fats, sweets, pastries, and sweetened carbonated and non-carbonated drinks.

Higher scores were given to responses indicating healthy dietary habits and lower points were given to responses indicating unhealthy dietary habits. For “adherence to a healthy diet”, summing up the answers formed a raw score, which we then scaled into a T score (standardized) with an average of 49.78 and standard deviation of 6.6 (min = 22, max = 66). Scores below 45 indicated adherence to an unhealthy diet and those above 56 indicated maximum values for adherence to a healthy diet (Table 2).

The majority of the respondents had a moderately healthy diet (56.21%), and 24.75% of them reported an increased adherence to a healthy diet. Among those with high adherence to a healthy diet, 81.68% were women (χ^2^ = 2.55, *p* = 0.279), most of whom (χ^2^ = 36.941, *p* < 0.001) were over 45 (34.43%) and very few were under 25 (15.75%). Most of them 82.05% came from urban areas (χ^2^ = 26.39, *p* = 0.471), 88.64% had higher education (χ^2^ = 26.08, *p* < 0.001), 63% were physically active (χ^2^ = 13.06, *p* = 0.220), 56.41% had a normal weight (χ^2^ = 12.515, *p* = 0.051), and 57.14% of them declared that they get urinary infections very rarely while 29.30% never did (χ^2^ = 13.46, *p* = 0.036). In the group that reported adherence to an unhealthy diet, the young population (up to 35 years old) and people from rural areas predominated, probably because the level of income can influence the possibility of purchasing nutritious food, and the highest percentage of respondents who stated that they frequently get urinary infections can be found in this group. The data presented in Table 2 indicate the lowest percentage of people over 45 years old (χ^2^ = 36.941, *p* < 0.001) was in the group of respondents with an unhealthy diet (14.76%) and the highest percentage was in the group of respondents with the highest adherence to a healthy diet (34.43%), which means that in our group of respondents, over 45% of people are more attentive to their food choices, consuming more nutritious foods and foods with protective factors (antioxidants, vitamins, minerals, prebiotics, probiotics).

Multiple linear regression analysis was used to investigate the association between adherence to a healthy diet (dependent variable), socio-demographic and anthropometric variables, and frequency of urinary infections (Table 3).

As the age of the participants increases, the tendency to adhere to an unhealthy lifestyle decreases (age group 18–23: OR = 3.79, 95% (CI): 2.00–7.14; age group 24–35: OR = 3.61, 95% (CI): 1.99–6.53; age group 35–45: OR = 2.67, 95% (CI): 1.49–4.79). Also, young people have the highest risk of adhering to a moderately healthy diet (age group 18–23: OR = 2.32, 95% (CI): 1.20–4.47).

Rural participants have a higher risk of adhering to an unhealthy diet than urban participants (OR = 1.84, 95% (CI): 1.03–2.92).

Participants with general/primary education (OR = 3.63, 95% (CI): 1.03–7.74) and those with secondary education (OR = 1.94 95% (CI): 1.05–3.60) have an increased tendency towards an unhealthy diet compared to participants with higher education.

Underweight participants have an increased risk of having an unhealthy diet (OR = 2.94 95% (CI): 1.37–6.31, *p* = 0.006) compared to normal-weight respondents.

Respondents who have urinary infections very often have a higher risk of adhering to an unhealthy diet (OR = 2.92, 95% (CI): 1.14–6.48), and those who state that they have relatively frequent urinary infections have a higher risk of adhering to a moderately healthy diet (OR = 2.04, 95% (CI): 1.20–3.45).

**Figure 5:**
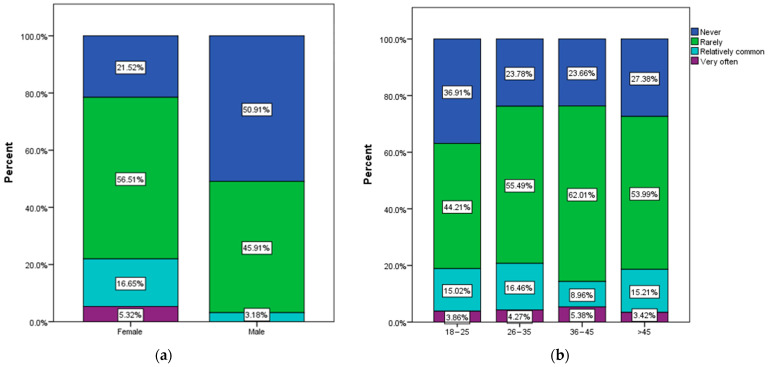
Frequency of urinary infections by (**a**) sex, (**b**) age.

A significant association was noted between sex (χ^2^ = 43.79, *p* < 0.001), age (χ^2^ = 26.47, *p* = 0.02), and the frequency of urinary infections (Figure 5).

Only female respondents declared that they frequently have urinary infections. Also, while 21.52% of female respondents declared that they never had urinary infections, the same statement applied to over 50% of male respondents. In terms of age, 26–35-year-olds were the most likely to declare that they frequently get urinary infections; they were also the least likely to report the absence of urinary infections. Respondents from the over 45 age group had a slightly lower predisposition to recurrent urinary infections compared to respondents from the 18–35 age group, but they also had a higher adherence to a healthy diet compared to them (Figure 5b).

**Figure 6:**
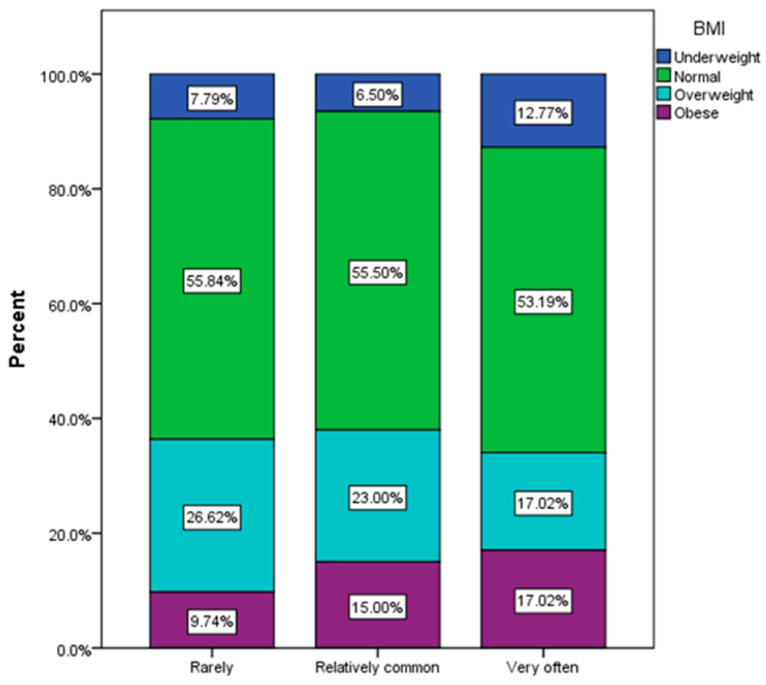
Frequency of urinary infections by BMI group.

According to the processed answers, it was found that in the group of obese respondents (Figure 6), the majority faced recurrent infectious episodes (χ^2^ = 21.14, *p* = 0.016).

**Figure 7:**
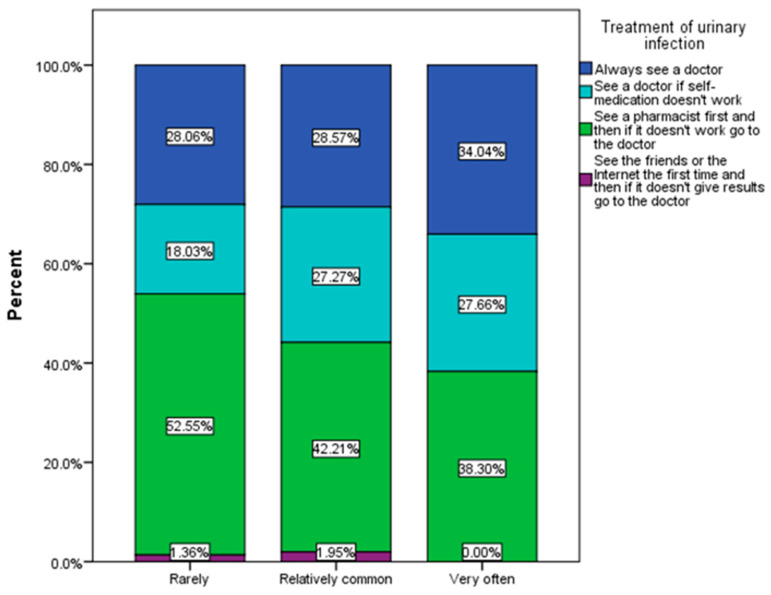
Therapeutic approach to urinary infections (χ^2^ = 20.31, *p* = 0.048).

As can be seen in Figure 7, a fairly large percentage of respondents said they usually go to the pharmacy first to treat urinary infections, and approximately 27% of those who have relatively frequent urinary infections go to the doctor after self-medication has had no effect (χ^2^ = 22.19, *p* = 0.014).

**Figure 8:**
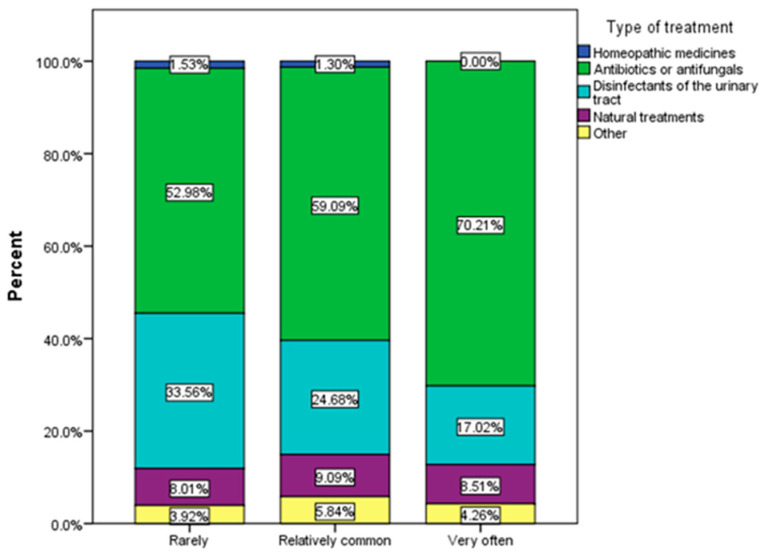
Type of treatment by frequency of urinary infections.

The most frequently used treatment in recurrent urinary infections were antibiotic and antifungal substances (Figure 8).

**Figure 9:**
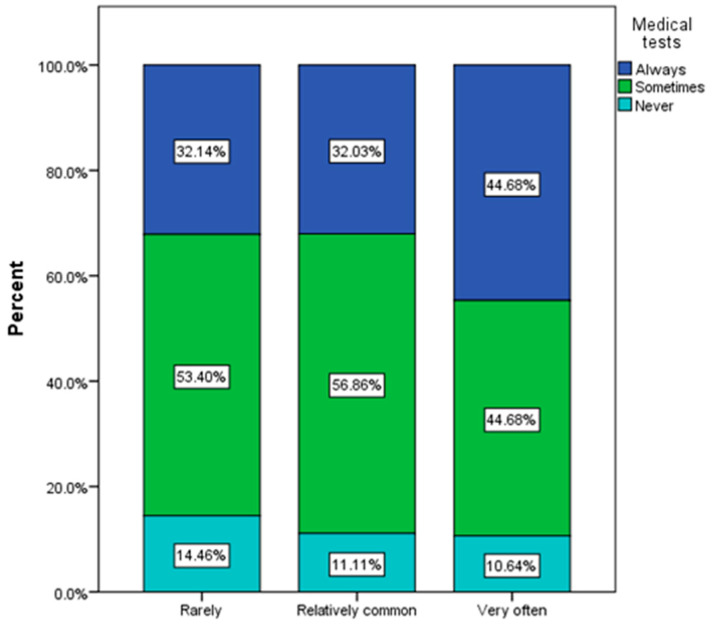
The use of medical tests (antibiograms) to identify the cause of urinary infection by frequency of urinary infections.

Those who most often used antibiograms to determine treatment with antibiotics were those who frequently have urinary infections (χ^2^ = 28.54, *p* = 0.002). In general, respondents declared that they sometimes used antibiograms as part of the therapeutic approach to infection (Figure 9).

**Figure 10:**
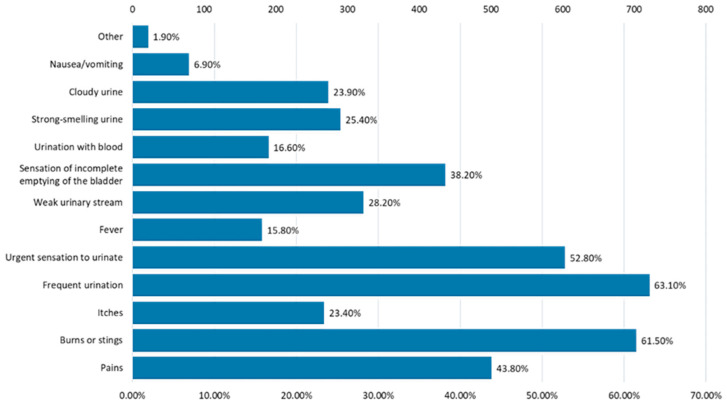
Symptoms of urinary infection episodes.

The most common symptoms of urinary infections were frequent urination, burning or stinging, the feeling of frequent urination, and pain (Figure 10).

**Figure 11:**
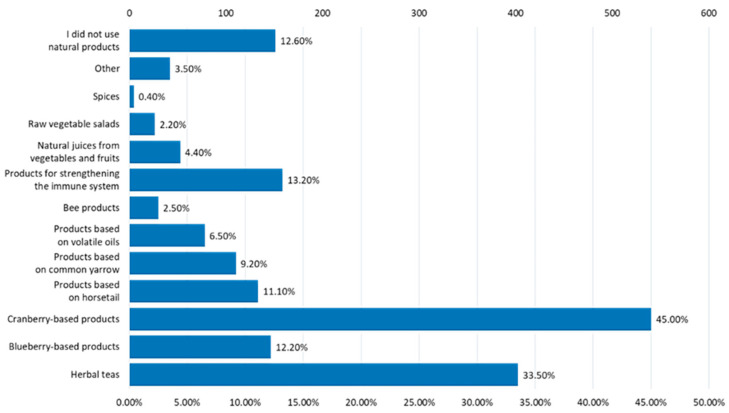
Types of natural treatments used for urinary infections.

The most frequently used natural treatments for urinary infections were cranberry-based products and herbal teas (Figure 11).

**Figure 12:**
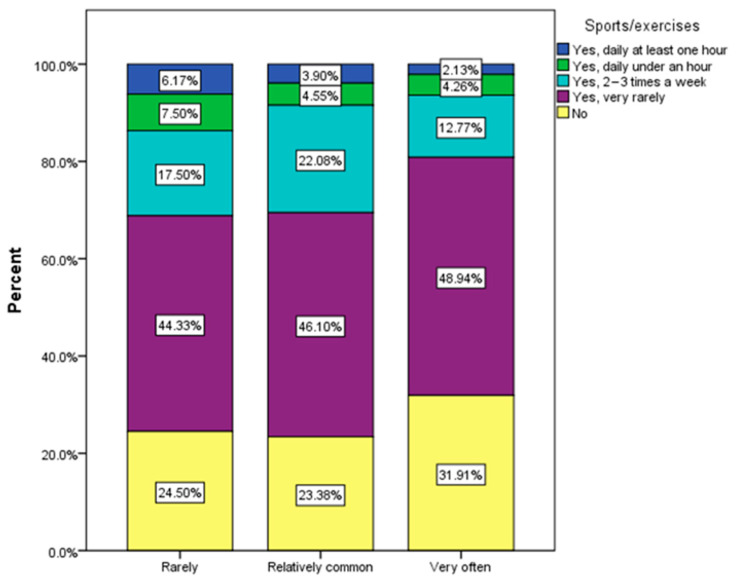
Sport activity and frequency of urinary infections (χ^2^ = 27.25, *p* = 0.005).

Sedentary respondents were more prone to frequent urinary infections. Physical activity improves the body’s detoxification and stimulates blood and lymphatic circulation (Figure 12).

## 4. Discussion

Based on this cross-sectional observational study in which 1103 volunteer respondents participated (Table 1), the majority whom were women (883) from urban areas (873), it was found through statistical processing of the collected data that recurrent urinary infections are influenced by a series of behavioral risk factors, mainly nutrition, hydration, physical activity, but also the therapeutic approach.

A total of 18.2% of the respondents, mainly women aged between 26 and 45 years, declared that they frequently experience urinary infections (Figure 3 and Figure 5), and 27.4% of the respondents declared that they had a urinary infection episode at least once in the last 6 months. There are clinical studies that draw attention to the seriousness of recurrent urinary tract infections, which can increase the risk of premature death even among young women [54,55]. According to epidemiological data, women are much more prone to urinary tract infections [56,57].

To analyze the influence of food on the risk of frequent development of urinary infections, adherence to a healthy diet was calculated and it was found that people who frequently consume nutritious foods that are rich in protective factors (prebiotics, probiotics, antioxidants, vitamins, minerals), hydrate effectively, and have a normal body weight have a significantly lower tendency to develop urinary infections frequently (Table 3). Also, following the statistical processing of the data, a greater tendency towards a healthy diet was noted among older people, those with higher education, and those with residence in urban areas (Table 3).

Among the main deficiencies relate to dietary habits found among the respondents participating in the study we noted the following: low consumption of fruits and vegetables (42.9% consumed a single portion of approximately 100 g of vegetables per day, 12% rarely consumed vegetables, 43.3% consumed a single portion of approximately 100 g of fruit per day, 15.1% consumed fruit very rarely); low consumption of fish or seafood; low consumption of foods rich in nutraceuticals (49.3% consumed them very rarely or not at all); low consumption of whole grains (6.2% consumed them daily); relatively high consumption of carbonated or sweetened non-alcoholic drinks (only 37.9% of respondents declared that they consume them very rarely or not at all); and a relatively high consumption of sweets and pastries (only 17.8% stated that they consume them very rarely or not at all). There was also a fairly large percentage of people who do not hydrate effectively; 31.5% of respondents stated that they usually consume about 1 L of water per day, and 10.1% just below 1 L. While 67.7% of the respondents declared that the main liquid consumed daily was plain water, there were also people who consumed coffee (16.9%) or carbonated or sweetened non-alcoholic drinks (12.3%) daily. There were also some positive aspects: a moderate consumption of bread, dairy products, eggs, alcoholic beverages, and fried foods, and the predominant consumption of food prepared at home. Numerous studies draw attention to protective factors in functional foods with a positive impact on the health of the intestinal microbiome, immunity, and the prevention of urogenital infections [58,59,60].

Regarding behavioral risk factors, an increased tendency towards sedentarism was found: 24.2% of the respondents declared that they do not usually engage in physical activity, 42.2% do sports very rarely, only 7.4% do sports daily for under an hour, and 6.6% do it daily for minimum one hour. This increased tendency towards sedentism explains the fact that the proportion of normal weight among the respondents is below 50% (Table 1). The lack of physical activity also influences the efficient detoxification of the body, the optimal oxygenation of the body, and the consumption of excess caloric food. Specialists have drawn attention to the importance of physical activity in the treatment and prevention of various ailments and to its multiple health benefits [61].

Another positive aspect found is the low tendency for smoking among the respondents: 68.8% were non-smokers and only 17.7% smoked excessively daily.

There are also negative aspects regarding the therapeutic approach to urinary infections: 25.6% of respondents use clinical investigations to identify urinary infections, 38.9% do so only sometimes, and 9.9% never. Regarding using an antibiogram to inform the choice of medicinal substance for treatment, approximately 22% turn to it every time. In the specialized literature, attention has been drawn to the need for a correct approach to the therapeutic plan associated with urinary tract infections [62,63].

Out of the total number of respondents, 27.4% answered that they did not have urinary infections; the majority of these (approximately half of them) were young, male respondents. This also explains the lower interest of men in voluntary participation in this study. This study was disseminated mainly online and through institutional emails (mainly university). The rural population either did not have access to the questionnaire, or spent much less time on social networks.

The limits of this study are the low participation of male respondents and of the rural population, but also of people over 45 years old. This study only analyzed the involvement of behavioral and dietary risk factors in the recurrence of urinary infections, not the associated pathologies. The importance of this study lies in the identification of behavioral risk factors that can significantly influence the recurrence of urinary infections and that must be addressed for effective prevention.

UTIs exhibit a marked prevalence among women compared to men. Empirical data demonstrate that approximately 50–60% of females will experience at least one UTI during their lifespan, whereas the incidence among males is notably lower, estimated at approximately 12% [64]. This variance primarily stems from anatomical distinctions; the comparatively shorter length of the female urethra facilitates a more direct passage for bacteria, thereby increasing the susceptibility to bladder infections. This explains the low interest of male respondents in participating in the study.

The processing of the questionnaire data highlighted an increase in the vulnerability to urinary infections among obese people, those who consume low amounts of fruit and vegetables, as well as those who are sedentary.

For the effective management of recurrent urinary infections, but also for optimal prevention, it is recommended to increase the consumption of fruit and vegetables, effective hydration, physical activity, and the correct therapeutic approach with the help of specialists in the medical field and based on specific clinical tests.

## 5. Conclusions

Food, hydration, and physical activity are the main behavioral factors that influence the recurrence of urinary infections, along with the therapeutic approach. A high-quality diet is rich in protective factors (fibers, antioxidants, vitamins, and mineral salts) that help strengthen the immune system and aid in detoxification; optimal hydration helps to effectively eliminate toxins from the body; and physical activity stimulates lymphatic circulation. This study shows a close relationship between these factors and the frequency of urinary infections. Also, the correct therapeutic approach, with the help of a specialist and based on the appropriate tests for the effective choice of medication, is crucial.

According to the results of this study, the most dangerous behavioral risk factors are related to excessive food consumption and sedentarism (obesity), the consumption of unhealthy food, and, especially, the reduced consumption of fruit and vegetables.

## Figures and Tables

**Table 1:** Socio-demographic and anthropometric characteristics of the respondents.

	Total Population		Female		Male	
	n	%	n	%	n	%
	1103	100	883	80.05	220	19.95
Age (years) (χ^2^ = 15.45, *p* < 0.001)						
18–25	233	21.12	196	22.20	37	16.82
26–35	328	29.74	275	31.14	53	24.09
36–45	279	25.29	222	25.14	57	25.91
>45	263	23.84	190	21.52	73	33.18
Residence area (χ^2^ = 10.089, *p* = 0.001)						
Urban area	873	79.15	716	81.09	157	71.36
Rural area	230	20.85	167	18.91	63	28.64
Level of education (χ^2^ = 15.529, *p* = 0.004)						
General/primary school	29	2.63	20	2.27	9	4.09
Secondary education (baccalaureate)	181	16.41	128	14.50	53	24.09
Postsecondary school	124	11.24	105	11.89	19	8.64
Higher education (bachelor’s degree)	412	37.35	339	38.39	73	33.18
Postgraduate studies (master’s degree, residency, doctorate, other specializations)	347	32.37	291	32.96	66	30.00
Employment status (χ^2^ = 17.87, *p* = 0.013)						
Unemployed	5	0.45	4	0.45	1	0.45
Socially assisted	3	0.27	1	0.45	2	0.23
Homemaker	37	3.35	29	3.28	8	3.64
Retired	30	2.72	18	2.04	12	5.45
Student	228	20.67	200	22.65	28	12.73
Teleworking	31	2.81	76	8.61	19	8.64
I go to work every day	674	61.11	528	59.80	146	66.36
I work in a hybrid regime (telework and commuting)	95	8.61	49	7.90	27	9.89
Body mass index (BMI) (χ^2^ = 32.46, *p* < 0.001)						
Underweight	80	11.43%	80	9.06	0	0.00
Normal weight	598	46.67%	547	61.95	51	23.18
Overweight	265	24.76%	158	17.89	107	48.64
Obese	160	17.14%	98	11.10	62	28.18

**Table 2:** Adherence to healthy diet by age group, gender, BMI group, residence area, education level, employment status, and frequency of urinary infection with the test of equality for column proportions (z-test).

Variable	Adherence to a Healthy Diet					
	Mean = 49.82, SD = 6.06, Min = 22, Max = 66					
	Unhealthy Diet (A)		Moderately Healthy Diet (B)		Healthy Diet (C)	
	n	%	n	%	n	%
Total	210	19.04	620	56.21	273	24.75
Gender (χ^2^ = 2.55, *p* = 0.279)						
Female	160	76.19	500	80.65	223	81.68
Male	50	23.81	120	19.35	50	18.32
Age (χ^2^ = 36.941, *p* < 0.001)						
18–25	63 ^B,C^	30.00	127	20.48	43	15.75
26–35	61	29.05	201 ^C^	32.42	66	24.18
36–45	55	26.19	154	24.84	70	25.64
>45	31	14.76	138	22.26	94 ^A,B^	34.43
Residence area (χ^2^ = 26.39, *p* = 0.471)						
Urban area	139	66.19	510 ^A^	82.26	224 ^A^	82.05
Rural area	71 ^B,C^	33.81	110	17.74	49	17.95
Level of education (χ^2^ = 26.08, *p* < 0.001)						
General/primary school	13 ^B,C^	6.19	12	1.94	4	1.47
Secondary education (baccalaureate)	50 ^C^	23.81	104 ^C^	16.77	27	9.89
Postsecondary school	22	10.48	64	10.32	38	13.92
Higher education (bachelor’s degree)	88	41.90	223	35.97	101	37.00
Postgraduate studies (master’s degree, residency, doctorate, other specializations)	37	17.62	217 ^A^	35.00	103 ^A^	37.73
Employment status (χ^2^ = 13.06, *p* =0.220)						
Unemployed	3	1.43	1	0.16%	1	0.37
Socially assisted	0	0.00	2	0.32	1	0.37
Homemaker	6	2.86	24	3.87	7	2.56
Retired	3	1.43	17	2.74	10	3.66
Student	57 ^C^	27.14	122	19.68	49	17.95
Teleworking	3	1.43	22	3.55	6	2.20
I go to work every day	119	56.67	383	61.77	172	63.00
I work in a hybrid regime (telework and commuting)	19	9.05	49	7.90	27	9.89
Body mass index (BMI) (χ^2^ = 12.515, *p* = 0.051)						
Underweight	24 ^C^	11.43%	44	7.10%	12	4.40%
Normal weight	98	46.67%	346	55.81%	154	56.41%
Overweight	52	24.76%	144	23.23%	69	25.27%
Obese	36	17.14%	86	13.87%	38	13.92%
Frequency of urinary infection (χ^2^ = 13.46, *p* = 0.036)						
Very often	15	7.14%	22	3.55%	10	3.66%
Relatively common	24	11.43%	103^C^	16.61%	27	9.89%
Rarely	115	54.76%	329	53.06%	156	57.14%
Never	56	26.67%	166	26.77%	80	29.30%

Columns with different uppercase letters (A, B, C) are statistically different (*p* < 0.05). SD = standard deviation.

**Table 3:** Results of multinomial logistic regression for adherence to a healthy diet.

Independent Variables	Unhealthy Diet			Moderately Healthy Diet		
	OR	95% CI	*p*-Value	OR	95% CI	*p*-Value
Gender						
Male	1			1		
Female	0.619	(0.363–1.055)	0.078	0.822	(0.538–1.256)	0.365
Age (years)						
18–23	3.789	(2.004–7.167)	<0.001	2.321	(1.204–4.476)	0.012
24–35	3.615	(1.994–6.535)	<0.001	2.220	(1.464–3.367)	<0.001
35–45	2.674	(1.491–4.795)	0.001	1.605	(1.067–2.414)	0.023
>45	1			1		
Residence area						
Urban area	1			1		
Rural area	1.849	(1.037–2.922)	0.008	0.889	(0.601–1.314)	0.554
Level of education						
General/primary school	3.635	(1.037–7.743)	0.044	1.558	(0.465–5.220)	0.473
Secondary education (baccalaureate)	1.949	(1.054–3.606)	0.033	1.887	(1.111–3.206)	0.019
Postsecondary school	0.780	(0.415–1.466)	0.440	0.832	(0.514–1.348)	0.455
Higher education (bachelor’s degree)	1			1		
Postgraduate studies (master’s degree, residency, doctorate, other specializations)	0.395	(0.239–0.653)	<0.001	0.879	(0.618–1.251)	0.585
Body mass index (BMI)						
Underweight (<18.5)	2.944	(1.372–6.318)	0.006	1.494	(0.759–2.941)	0.245
Normal (18.5–24.9)	1			1		
Overweight (25–29.9)	1.275	(0.773–2.104)	0.091	0.983	(0.672–1.438)	0.929
Obese (≥30)	1.351	(0.753–2.423)	0.313	1.081	(0.684–1.707)	0.739
Frequency of urinary infection						
Very often	2.925	(1.143–6.488)	0.025	1.165	(0.514–2.639)	0.715
Relatively common	1.719	(0.856–3.455)	0.128	2.043	(1.208–3.454)	0.008
Rarely	1.417	(0.898–2.235)	0.134	1.071	(0.758–1.514)	0.698
Never	1			1		

Dependent variable: healthy diet as the reference category.

## Data Availability

Data is contained within the article.
